# The profile of patients with postpartum hemorrhage admitted to the obstetric intensive care: a cross-sectional study

**DOI:** 10.61622/rbgo/2024rbgo47

**Published:** 2024-06-27

**Authors:** Mayara dos Santos Farias Ferreira Silva, Melania Maria Ramos de Amorim, Brena Melo, André Vieira Lanza, Maria Eduarda Trigueiro Ramos, Bruna Antunes Durães de Carvalho, Natalia Nunes Tenório, Leila Katz

**Affiliations:** 1 Instituto de Medicina Integral Prof. Fernando Figueira Recife PE Brazil Instituto de Medicina Integral Prof. Fernando Figueira, Recife, PE, Brazil.; 2 Universidade Federal e Campina Grande Campina Grande PB Brazil Universidade Federal e Campina Grande, Campina Grande, PB, Brazil.; 3 Teaching Hospital Universidade Federal de Uberlândia Uberlândia MG Brazil Teaching Hospital, Universidade Federal de Uberlândia, Uberlândia, MG, Brazil.; 4 Faculdade Pernambucana de Saúde Recife PE Brazil Faculdade Pernambucana de Saúde, Recife, PE, Brazil.

**Keywords:** Postpartum hemorrhage, Maternal mortality, Pregnancy, Intensive care units

## Abstract

**Objective:**

In Brazil, postpartum hemorrhage (PPH) is a major cause of maternal morbidity and mortality. Data on the profile of women and risk factors associated with PPH are sparse. This study aimed to describe the profile and management of patients with PPH, and the association of risk factors for PPH with severe maternal outcomes (SMO).

**Methods:**

A cross-sectional study was conducted in Instituto de Medicina Integral Prof. Fernando Figueira (IMIP) obstetric intensive care unit (ICU) between January 2012 and March 2020, including patients who gave birth at the hospital and that were admitted with PPH to the ICU.

**Results:**

The study included 358 patients, of whom 245 (68.4%) delivered in the IMIP maternity, and 113 (31.6%) in other maternity. The mean age of the patients was 26.7 years, with up to eight years of education (46.1%) and a mean of six prenatal care. Uterine atony (72.9%) was the most common cause, 1.6% estimated blood loss, 2% calculated shock index (SI), 63.9% of patients received hemotransfusion, and 27% underwent hysterectomy. 136 cases of SMO were identified, 35.5% were classified as maternal near miss and 3.0% maternal deaths. Multiparity was associated with SMO as an antepartum risk factor (RR=1.83, 95% CI1.42-2.36). Regarding intrapartum risk factors, abruptio placentae abruption was associated with SMO (RR=2.2 95% CI1.75-2.81). Among those who had hypertension (49.6%) there was a lower risk of developing SMO.

**Conclusion:**

The principal factors associated with poor maternal outcome were being multiparous and placental abruption.

## Introduction

Worldwide, almost 14 million women annually develop increased bleeding during delivery or postpartum and more 80,000 women die as a result of postpartum hemorrhage (PPH).^(1-3)^ Over 90% of these deaths should not have occurred^(4)^ and are classified as avoidable by the World Health Organization (WHO).^(2)^

Identifying the factors that increase the risk of PPH should be part of the care provided to pregnant women, beginning with prenatal care but, principally, at admission to maternity hospital and continuously during labor and childbirth as a step in the prevention and management of this event.^(5)^ Patients with risk factors deserve specific attention and care to prevent hemorrhage and severe secondary complications; however, 20% of cases of PPH occur even in the absence of any prior risk factor.^(6-11)^

Management of PPH is successful when the condition is identified early, and opportune treatment is directed towards specific causes. Correctly estimating blood loss allows the appropriate diagnosis to be made and timely treatment implemented.^(9)^ There is a direct association between severe maternal outcome (SMO) and the time between diagnosis and treatment of PPH. Controlling the cause of bleeding as early as possible is the most effective means of combatting an SMO associated with massive blood loss.^(12)^

Identifying the cause of PPH is crucial, with the principal causes being the 4 T’s of PPH: tone, trauma, tissue and thrombin.^(6-10,13)^ Pharmacological treatment is essential in managing PPH, since the principal cause by far is uterine atony. Uterotonic regimens are varied and should be used sequentially until bleeding is controlled.^(3,5,14)^

Interventions recommended by the WHO for the treatment of PPH include fluid replacement, the early use of tranexamic acid and nonsurgical measures such as uterine balloon tamponade, non-pneumatic anti-shock garments and external aortic compression. Finally, if the bleeding persists, opportune surgical treatment (compression sutures, arterial ligation, hysterectomy, damage control surgery) should be implemented; however, as a rule, this should be an alternative only when all other strategies fail.^(6-11,13,15)^

The prevention, diagnosis and appropriate treatment of PPH represent crucial steps in implementing actions for the care of women during labor, childbirth and postpartum to promote improvements in women’s health and achieve the sustainable development goal to reduce maternal mortality.^(1,2,4)^ Therefore, the objective of this study was to describe the profile of patients diagnosed with PPH in the obstetric intensive care unit (ICU) of the *Instituto de Medicine Integral Prof. Fernando Figueira* (IMIP), the management of cases, their outcomes and the association between the presence of risk factors for PPH and SMO.

## Methods

This cross-sectional study is an arm of a retrospective cohort study entitled: “Postpartum hemorrhage in an obstetric ICU in northeastern Brazil: a retrospective cohort study”. A secondary analysis was conducted of the database constructed for that previous study. The study was developed in the ICU of the Women’s Healthcare Center, IMIP in Recife, Pernambuco, northeastern Brazil. Patients admitted to the obstetric ICU with a diagnosis of PPH between January 2012 and March 2020 were included in the study.

Near miss maternal mortality (NMM) was defined according to the WHO criteria,^(16)^ and likewise, SMO was defined as the sum of the number of deaths and maternal near misses.^(17)^

For this particular analysis, sample size was not calculated, since all the patients who met the eligibility criteria during the study period were included. The records were evaluated and the data of interest extracted. Patients whose diagnosis of PPH was ruled out after their hospital records were examined were excluded from the study.

Statistical analysis was conducted using Epi Info, version 7.2.5.0. Initially, frequency distribution tables were obtained for the categorical variables and measures of central tendency and dispersion were calculated for the numerical variables. To compare numerical variables between the two groups, Student’s t-test was used for the continuous variables with normal distribution and the Mann-Whitney test for non-normal variables (discrete or ordinal data). The categorical variables were compared in contingency tables using Pearson’s chi-square test of association or Fisher’s exact test, as pertinent. Bivariate analysis was performed to verify the existence of an association between the presence or absence of risk factors for PPH and the presence of SMO using Pearson’s chi-square test of association or Fisher’s exact test, as appropriate. The significance level adopted was 5%. To determine the strength of the association, relative risks (RR) and their 95% confidence intervals (95%CI) were calculated. Missing data was excluded from the analysis of each variable.

IMIP’s internal review board approved the study protocol under reference (5.402.144) CAAE 58449822.2.0000.5201. Requirement for informed consent was waived.

## Results

**Figure 1:**
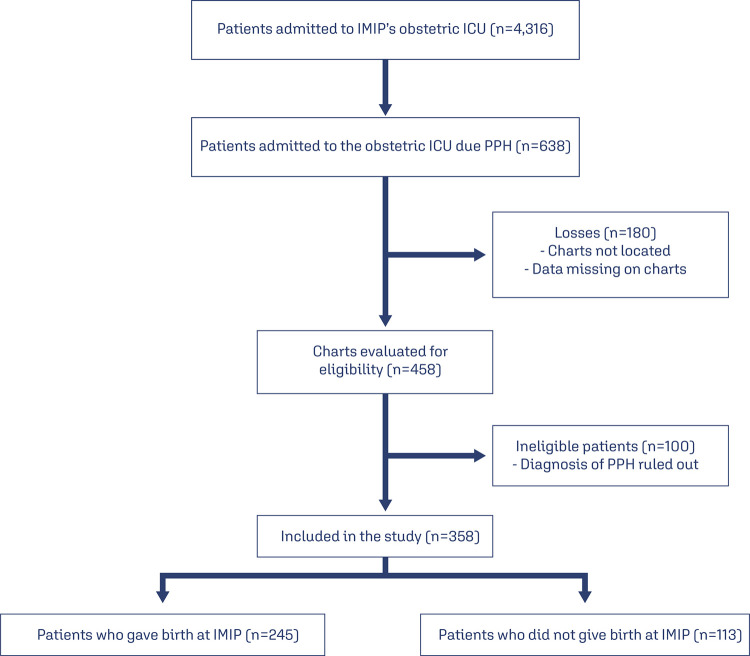
Data collection process

Of the 4,316 women admitted to IMIP’s ICU during the study period, 608 were diagnosed with PPH according to the sector’s database. Of these, 458 charts were retrievable; however, 100 of these were excluded either because their diagnosis of PPH was ruled out after their hospital charts had been examined, or because their records were insufficient for analysis, resulting in a total of 358 patients. Of these, 245 (68.4%) delivered at IMIP’s maternity unit, while 113 (31.6%) had been referred from other maternity hospitals after delivery (Figure 1).

Patients ranged from 12 to 46 years of age (mean 26.7 years). Those under 19 years of age corresponded to 19.5% of the sample, while 15.4% were over 35 years of age. Regarding ethnicity/skin color, 70.2% were brown-skinned, 22% white and 5.7% were black (Table 1).

**Table 1:** Socioeconomic characteristics of the study population and the number of prenatal visits they attended

Variables	n(%)
Age	
Mean / standard deviation	26.7(7.6)
Minimum/maximum	12(46)
<19 years	70(19.5)
>35 years	55(15.4)
Skin color/ethnicity *	
White	31(22)
Brown	99(70.2)
Black	8(5.7)
Yellow	3(2.1)
Indigenous	0
Education level ^§^	
1^st^ to 4^th^ grade	13(6)
5^th^ to 8^th^ grade	88(41.1)
High school	100(46.7)
Some university education	2(0.9)
Graduated from university	11(5.1)
Place of residence (n/%) ^˥^	
Recife metropolitan region	166(50.3)
Elsewhere in Pernambuco state	161(48.8)
Other states	3(0.9)
Marital status ^¥^	
Single	75(32.5)
Married	80(34.6)
In a stable union	75(32.5)
Widowed	1(0.4)
Number of pregnancies ^¶^	
Median / interquartile range (IQR)	2(1-3)
Minimum/maximum	1(1-2)
Number of deliveries ^¶^	
Median / IQR	1(1-2)
Minimum/maximum	0(10)
Number of prenatal visits ^ǂ^	
Median / IQR	6(4-7)
None (n/%)	2(1.27)
< 6 visits (n/%)	76(48.1)
≥ 6 visits (n/%)	82(51.9)
Cause of PPH (n/%) ^ǀ^	
Hypotonia/atony	261(72.9)
Tear	48(13.4)
Retained products of conception	52(14.5)
Coagulopathy ^ǁ^	12(3.3)

^*^ Information available for only 141 cases; ^§^ Information available for 214 cases; ^˥^ Information available for 330 cases; ^¥^ Information available for 231 cases; ^¶^ Information available for 357 patients, since one patient was admitted in a severe condition following Cesarean section in another institute and no information was available on parity; ^ǂ^ Information available for 159 cases; ^ǀ^ Each patient can have more than one cause of bleeding; ^ǁ^ Including primary coagulopathies as well as patients who developed coagulopathies secondary to other diseases such as placental abruption, sepsis and steatosis, and for that reason developed PPH

Data on education level had been recorded on 214 charts. Of these, 13 women (6%) had 1-4 years of schooling, while 88 (41.1%) had 5-8 years of schooling, 100 (46.7%) had completed high school, 2 (0.9%) had some university education and 11 (5.1%) had graduated from university. Regarding marital status, 34.6% of the women were married, 32.5% were in a stable union and a similar percentage was single. Fifty percent of the women lived in the metropolitan region of Recife, 48.8% in another town in the same state, and 0.9% lived in another state (Table 1). Participants had had a median of two pregnancies (interquartile range [IQR] 1-3), with a median of 1 delivery (IQR 1-2). Analysis of the number of prenatal visits made by the patients admitted to the ICU showed that this information was available for only 159 cases. The median number of visits was 6 (range 4-7). In 51.9% of cases, the women had attended six or more prenatal consultations, while 48.1% of the women had attended fewer than 6 prenatal visits and 1.3% had not attended any prenatal visits. The principal cause of PPH in these women admitted to the ICU was uterine atony, recorded in 72.9% of cases, followed by retained products of conception in 14.5%, perineal laceration in 13.4% and coagulopathy in 3.3% of cases (Table 1). There could have been more than one cause of PPH per patient. In relation to the characteristics of the management of PPH, blood loss was quantified for only 1.6% of all the patients who had PPH at IMIP, with the method used being visual calculation in all cases. The shock index was calculated and described on the charts in only 2% of cases. In 34.3% of cases, uterine massage performed to treat uterine atony was described on the charts. Analysis of the use of uterotonic drugs to treat PPH showed that oxytocin was the drug of choice in 87.7% of cases, misoprostol in 66.9%, ergometrine in 17.1% and tranexamic acid in 19.6% of cases. One single patient could have received more than one drug. The volume of crystalloid fluid used in hypovolemic resuscitation was recorded in 111 cases, with the volume instilled ranging from 500 to 9,000 ml. The mean volume of crystalloid fluid was 2,500 ml (Table 2).

**Table 2:** Characteristics of the management of hemorrhage (data available for the 245 patients whose delivery occurred at IMIP)

Characteristics	n(%)
Visually estimated blood loss	4(1.6)
Calculation of Shock Index	5(2)
Volume of crystalloid fluid used in fluid resuscitation in mL(median /IQR)*	2500(1500-3000)
Description of uterine massage for the treatment of atony	84(34.3)
Description of oxytocin use	215(87.7)
Description of misoprostol use	164(66.9)
Description of ergometrine use	42(17.1)
Description of tranexamic acid use	48(19.6)
Total	245(100)

* The volume of crystalloid fluid used for fluid resuscitation was recorded in 111 cases and ranged from 500 to 9000 ml

Regarding the management of bleeding, complications and maternal outcomes, 229 patients (63.9%) received blood transfusions to replace blood components; 41 patients (11.4%) received fresh plasma; 31 (8.6%) received platelet concentrate; and 5 (1.4%) received cryoprecipitate. As a complication of PPH, 32.9% of the patients developed hypovolemic shock. Analysis of invasive procedures showed that hysterectomy (27%) was the principal surgical procedure used to control PPH, followed by compressive sutures (22.8%) and arterial ligation (4.7%). Intrauterine balloons were used to manage PPH in only 1.1% of patients (Table 3).

**Table 3:** Characteristics of the management of hemorrhage, complications and outcome

Characteristics	n(%)
Transfusion of blood components	229(63.9)
Use of fresh plasma	41(11.4)
Use of platelet concentrate	31(8.6)
Use of cryoprecipitate	5(1.4)
Compressive sutures	62(22,8)
Hysterectomy	97(27)
Arterial ligation	17(4.7)
Intrauterine balloon	4(1.1)
Hypovolemic shock	118(32.9)
Coagulopathy	19(5.3)
Mechanical ventilation	26(7.3)
Dialysis due to acute kidney injury	10(2.8)
Use of vasoactive drugs	10(2.8)
Duration of time in intensive care (median/IQR)	3(2-4)
Near miss	125(34.9)
Death	11(3)

Of the patients admitted to the ICU, 7.3% were submitted to mechanical ventilation, while 2.8% received vasoactive drugs and 2.8% were submitted to dialysis for acute kidney injury (AKI). A total of 136 patients had an SMO (37.9%), 125 had a maternal near miss (34.9%) and 11 (3%) died (Table 3). The primary antepartum risk factors in patients admitted to the obstetric ICU with PPH were elevated blood pressure (45.8%), prior C-section (24.5%), and multiparity (20.6%). Intrapartum risk factors included placental abruption (11.7%) and induced labor (10.9%). These data pertain to all women admitted to the ICU, whether with or without SMO. For enhanced clarity and comparison, Table 4 displays these data categorized by the presence of SMO. Analysis of the association between the presence of risk factors and SMO showed that, of the reproductive characteristics, multiparity, present in 32.3% of the patients, was associated with SMO as an antepartum risk (RR=1.83; 95%CI: 1.42 - 2.36; p<0.01). In relation to the intrapartum risks, placental abruption was associated with SMO (RR=2.2; 95%CI: 1.75 - 2.81; p<0.01). The risk of SMO was lower (RR=0.70; 95%CI: 0.52 - 0.93; p=0.011) in the women who developed hypertension (49.6%) (Table 4).

**Table 4:** Association between risk factors and severe maternal outcome

Risk factors	Severe maternal outcome		RR	95%CI	p-value
	Yes n= 136 n(%)	No n= 222 n(%)			
Previous history					
Past history of PPH	3(2.2)	3(1.3)	1.32	0.58-2.97	0.67*
Congenital or acquired coagulation disorders	6(4.4)	5(2.2)	1.45	0.83-2.53	0.25
Use of anticoagulants	1(0.7)	2(0.9)	0.87	0.17-4.36	1.00*
Multiparous	44(32.3)	30(13.5)	1.83	1.42-2.36	<0.01
Previous Cesarean section	34(25.0)	54(24.3)	1.02	0.75-1.38	0.88
Previous Cesarean section with placenta previa	2(1.5)	4(1.8)	0.87	0.28-2.73	1.00*
Increased blood pressure	49(36.0)	115(49.6)	0.70	0.52-0.93	0.01
Anemia during pregnancy	5(3.7)	1(0.45)	2.2	1.52-3.28	0.03*
Uterine distension	5(3.7)	6(2.7)	1.2	0.62-2.33	0.83
Twin pregnancy	5(3.7)	16(7.21)	0.61	0.28-1.33	0.17
Polyhydramnios	5(3.7)	4(1.8)	1.4	0.81-2.69	0.30*
Intra-partum factors					
Third or fourth degree vaginal tear	2(1.5)	2(0.9)	1.32	0.49-3.55	0.36*
Placental abruption	31(22.8)	11(4.9)	2.22	1.75-2.81	<0.01
Placental abnormality	8(5.8)	5(2.2)	1.66	1.05-2.60	0.07
Induced delivery	8(5.8)	31(13.9)	0.51	0.27-0.96	0.01
Chorioamnionitis	3(2.2)	8(3.6)	0.71	0.26-1.88	0.54*
Instrumental delivery	1(0.7)	24(10.8)	0.09	0.01-0.67	<0.01

* Fisher’s exact test

## Discussion

These results define the profile of the women diagnosed with PPH who were admitted to an obstetric ICU in northeastern Brazil. This population consisted of young, brown-skinned, literate patients who lived in the major metropolitan regions of the state and had had a median of two pregnancies and attended a median of six prenatal visits.

Uterine atony was the principal cause of PPH, accounting for 72.9% of cases. This finding corroborates the results of other studies that reported rates ranging from 60 to 80%.^(6-8,18-22)^ Other causes included retained products of conception (14.4%), perineal laceration (13.4%) and coagulopathy (3.3%), with these rates also being in agreement with previously published data.^(19-22)^

According to the etiology of PPH, the principal pharmaceutical treatment consisted of uterotonic drugs. As expected and in accordance with WHO guidelines, injectable oxytocin was the principal drug used (87.7%),^(6-11,16,23)^ followed by misoprostol (66.9%), tranexamic acid (19.6%) and ergometrine (17.1%). The use of misoprostol as a second-line agent can be explained by the high prevalence of women with hypertensive complications associated with PPH, which contraindicates the use of ergometrine. Although the WHO recommends the use of tranexamic acid in the early management of postpartum hemorrhage, the low level of use may have been influenced by some factors, such as the use of sequenced treatment to control hemorrhage, as well as the possibility of underreporting the steps taken, which can happen in retrospective records, as has been reported in some studies.^(14)^

In the context of PPH management, blood loss was quantified in only 4 (1.6%) cases, exclusively through visual design, an inaccurate method that can delay or prevent the initiation of potentially life-saving interventions. The documentation of blood loss estimations took place in the delivery room. Challenges in adhering to institutional protocols for weighing compresses and fields to estimate loss, compounded by reliance on paper records and temporal degradation, suggest a potential limitation in consistently recording crucial information. The management predominantly relies on clinical parameters, highlighting the need for enhanced methodologies in capturing precise PPH-related data, such as a pre-designed chart containing key information about PPH.

Some studies have already reported results that contributed towards improving the quality of care in cases of PPH. In Wales, intrauterine balloons were used in 28.5% of cases, with hysterectomy accounting for only 4.6% and no deaths from PPH.^(24)^Another study conducted in Japan found an association between increased use of intrauterine balloons (20%) with a significant reduction in hysterectomy (6.2%) and a trend towards a reduction in PPH-related maternal mortality.^(20)^

In the present study, arterial ligation was performed in only 4.7% of patients and intrauterine balloon used in only 1.1%, rates that are lower than those reported in other studies.^(20,24)^ The most common surgical intervention was hysterectomy (27%), although approaches such as compression sutures (22.8%) and arterial ligation, associated or not with intrauterine balloons, allowed the uterus to be conserved without increasing morbidity. This may have occurred as a result of the patients’ ages or multiparity or the team’s lack of experience with the techniques. It is also possible that, since this was a group of more seriously ill patients requiring intensive care, hysterectomy may have been necessary and maintaining conservative forms of management may often have proven impossible, thus explaining the findings.

The mean age at delivery was 26.7 years, a finding that is in agreement with the results of other studies;^(18,19)^ however, the percentage of women under 19 years of age (19.5%) was higher than figures previously reported.^(19-22)^ This may reflect the characteristics of the region in which the study was performed and where the percentage of adolescent pregnancies is higher.^(25)^

In this study, 48.8% of the patients came from other cities and states and this is in agreement with another Brazilian study (52.6%).^(26,27)^ One explanation could be that this ICU is exclusively reserved for obstetric patients and is situated in a quaternary referral center for the whole state. This could delay management of the case and access to the service, resulting in more severe PPH and a risk of developing an SMO.

Around 50% of patients had attended fewer than six prenatal visits, which could have played a role in the occurrence of PPH and patient recovery. A study conducted in Gabon^(19)^ reported that 73.6% of patients with PPH in that study had attended only four prenatal visits, with the recovery of the patients with PPH who had been monitored during pregnancy being faster than that of the women who had not been monitored. This could be associated with prenatal iron supplementation and the identification of prenatal risk factors.^(21)^

Uterine massage was registered in only 34.4% of cases despite its use being recommended for the management of PPH resulting from uterine atony.^(22,28)^ Unfortunately, the patient charts did not attest to compliance with this recommendation, probably since this is a retrospective study and, also, because the techniques used may not always be recorded and data may be lost, hampering evaluation. This has already been reported in the Netherlands.^(29)^ where healthcare professionals recorded fewer cases than were actually performed. Studies conducted in Brazil have also described how inadequate completion of charts can compromise care and hamper data analysis.^(14)^

Of the 245 patients evaluated here, the shock index (SI) was recorded in only 5 cases (2%), despite the fact that shock (32,9%) was the most common complication. The SI is a useful clinical parameter, easily applied to determine hemodynamic conditions and prevent the need for blood transfusion or surgical intervention, allowing patients with hemodynamic disorders to be identified early.^(6-10)^ A cohort study conducted in Japan to investigate the effect of standardizing the initial treatment for PPH using objective criteria (the start of transfusion and transportation based on the SI) concluded that evaluating the SI can contribute towards reducing hysterectomy and maternal mortality rates.^(20)^

The low incidence of hypovolemic shock (32.9%) among the women included in the study could be attributed to the fact that some women had milder forms of PPH which did not lead to the development of hypovolemic shock and were admitted to the ICU due to hypertensive disorders. It could also be explained by the fact that a significant proportion of the women (48.8%) were referred from other institutions, which could indicate that these patients had already received some form of treatment or stabilization before being transferred. Another factor to consider is the quality and consistency of the clinical record, which can influence the assessment of the severity of cases. If the record does not adequately reflect the evolution of the condition, the analysis of the development of hypovolemic shock may be underestimated.

Mechanical ventilation due to bleeding complications was required in 7.3% of cases and the need for vasoactive drugs and dialysis due to AKI in 2.8%. These results may be due to the fact that many of the patients who developed severe morbidities suffered hypertensive disorders.^(26)^ It is common for patients with hypertensive disorders to be admitted to an obstetric ICU for monitoring and the associated PPH could be mild and without repercussions. This may be why hypertension proved to be a lower risk for SMO in this study.

A total of 127 women (36%) were classified as maternal near miss, which is a more sensitive indicator with which to evaluate the quality of obstetric care. In a multicenter, cross-sectional study, PPH accounted for 23.5% of cases of maternal near miss in Brazil.^(30)^ The high percentage found in the present study could be explained by the fact that all the patients were admitted to the obstetric ICU, i.e. their condition was more severe. Furthermore, a significant percentage of patients (48.8%) had been transferred from other hospitals, which could have delayed treatment and affected their prognosis.

The percentage of maternal death due to PPH in the obstetric ICU was 3.3%, a rate that is higher compared to other studies,^(20,21,30)^possibly due to the associated complications and comorbidities and the fact that severe cases were referred to this institute from the entire state.

In this study, multiparity was identified as a risk factor for the development of SMO. Multiparity is a known risk factor for PPH, as already reported in other studies.^(18-22)^ Increased parity is a risk factor for uterine atony, the principal cause of increased bleeding following childbirth, and for death due to PPH in Brazil.^(14)^

Another risk factor identified here that increased the rate of SMO was placental abruption, with a rate (22.8%) that is higher than rates reported from other studies (6.62% in Ethiopia^(21)^ and 5.4% in Gabon.^(19)^ Nevertheless, the percentage was similar to that reported in a Brazilian study that analyzed maternal mortality rates from hemorrhage and identified placental abruption (30%) as being one of the principal causes.^(14)^ The reasons given to explain the high rate of placental abruption were that it could be associated with the multifactorial nature of the event^(31)^or with previous Cesarean section rates (24.5%). Patients who developed placental abruption suffered bleeding prior to delivery and for this reason were more likely to develop PPH, both due to uterine atony and coagulopathy.

In this study, neither the presence nor the absence of anemia was identified as a risk factor for SMO although anemia during pregnancy has been described as a risk factor for increased bleeding, for a need for blood transfusion and for SMO in other studies.^(18,21,22,27)^ Evaluation of these and other risk factors was prevented due to missing data, since many patients had been transferred from other institutes and there was no information on their obstetric history or their delivery. However, the possibility of anemia cannot be ruled out, since anemia in pregnancy is common in Brazil and is a modifiable risk factor for blood transfusion.^(32)^

Since, at the time of this study, routine risk classification had not yet been implemented at this healthcare institute, an attempt was made to identify the risk factors described on the charts and to determine if an association existed between these risk factors and SMO. Had a risk classification system been used, it could have helped avoid adverse outcomes, since care could have been escalated and SMO prevented if risk factors such as previous anemia, multiparity and placental abruption had been identified.

A strongpoint of this study lies in the fact that there are still few studies in Brazil that have evaluated the association between the presence of risk factors for PPH and the presence of SMO. Furthermore, the sample consists of a significant number of patients with PPH admitted to the obstetric ICU representing the northeastern region of Brazil.

Possible limitations to take into consideration include the fact that hemorrhagic events tend to be recorded retrospectively and the sequence followed is not always described. This form of registration hampers analysis of the appropriateness of treatment and evaluation of the success of each step, which may indicate that the lack of consensus among professionals regarding how to identify and measure blood loss could interfere in the classification and management of the event. Nevertheless, these results are relevant and allow the identification of aspects requiring categorization and improvement in order to increase the usefulness of recorded information.

## Conclusion

Identifying risk factors, such as multiparity and placental abruption, is essential for preventing serious maternal outcomes. The retrospective recording of PPH can impact on the analysis of the adequacy of treatment and the lack of consensus among professionals regarding the measurement of blood loss can interfere with the early detection, management and prevention of adverse outcomes. The implementation of risk classification systems can be a valuable tool for optimizing clinical approaches and improving maternal outcomes.
